# Museum Genomics of an Important Swedish Pollinator Reveals Population Stability Across Time

**DOI:** 10.1111/mec.70557

**Published:** 2026-09-22

**Authors:** Michael Mitschke, Ellen Karlsson, Jovanka Suess, Ibai Ugarte‐Zabaleta, Kalle Tunström, Björn Cederberg, Hege Vårdal, Matthew T. Webster, Christopher W. Wheat, David Díez‐del‐Molino

**Affiliations:** ^1^ Centre for Palaeogenetics Stockholm University Stockholm Sweden; ^2^ Department of Zoology Stockholm University Stockholm Sweden; ^3^ Department of Zoology Swedish Museum of Natural History Stockholm Sweden; ^4^ Department of Population Analysis and Monitoring Swedish Museum of Natural History Stockholm Sweden; ^5^ Department of Biology Lund University Lund Sweden; ^6^ Swedish Species Information Centre Swedish University of Agricultural Sciences (SLU) Uppsala Sweden; ^7^ Department of Medical Biochemistry and Microbiology, Science for Life Laboratory Uppsala University Uppsala Sweden; ^8^ Science for Life Laboratory Solna Sweden; ^9^ Department of Bioinformatics and Genetics Swedish Museum of Natural History Stockholm Sweden

**Keywords:** biodiversity crisis, bumblebee, global change, historical DNA, insect decline, museomics, pollinator

## Abstract

Anthropogenic activity has driven a rapid decline in wild pollinator populations over the last several decades. Understanding the genomic consequences of these declines and their long‐term effects requires establishing historical baselines for population diversity. To investigate this, we conducted temporal genomic analyses of a widespread and important pollinator, the common carder bee ( *Bombus pascuorum* ). We sequenced 102 historical, dry‐pinned museum specimens collected across Sweden during the last 150 years, and analysed them alongside 59 modern individuals. While historical samples showed elevated levels of fragmentation with age (range 41.8–89 bp), they also had overall high levels of endogenous DNA (median 86.9%). Our analyses revealed that while geographically isolated populations in Gotland and the Arctic mountains exhibit localized genomic differentiation and lower diversity, the overall mainland population structure is low. We find that population structure has remained stable and genomic diversity has increased across Sweden over the past 150 years. These results challenge assumptions of decline, indicating that *B. pascuorum* has resisted genomic erosion even with reported declines in relative abundance. This study validates dry‐pinned museum insects as a powerful resource for large‐scale conservation genomics studies, providing a useful temporal perspective despite DNA degradation.

## 1 Introduction

Rapid anthropogenic global change has resulted in a biodiversity crisis affecting a wide range of taxa (Cowie et al. 2022; Barnosky et al. 2011). Insects are particularly affected and show substantial declines in distribution ranges (e.g., Warren et al. 2001), species richness (e.g., Dupont et al. 2011), and biomass (e.g., Hallmann et al. 2017), likely primarily driven by agricultural intensification (Cardoso et al. 2020; Wagner et al. 2021; Sánchez‐Bayo and Wyckhuys 2019). Insect declines pose a considerable risk to various ecosystem services (Noriega et al. 2018; Sánchez‐Bayo and Wyckhuys 2019), one of the most prominent being pollination, which is essential for agriculture and ecosystem functioning and has an estimated annual global worth of 235–577 billion US‐Dollar (IPBES 2016).

Pollination is more successful when the pollinator community is more diverse (Dainese et al. 2019); a decline in the diversity of wild pollinators can have negative effects on, for example, fruit set, seed set, and fruit weight (Artamendi et al. 2025). While there is strong evidence for declines of several pollinating taxa in recent decades, including, for example, in lepidopterans, hoverflies, and wild bees, such as bumblebees (Potts et al. 2010; Wagner et al. 2021; Sánchez‐Bayo and Wyckhuys 2019), there is generally only a limited availability of long‐term records and historical records of abundances and ranges. The quality of historical records is vastly different between taxa; butterflies, for example, are relatively well represented, while information on solitary bees is scarce. An alternative to relying solely on observational datasets is to harness the potential of historical DNA from museum specimens to study species declines and the effects of global change (Nakahama 2021; Díez‐Del‐Molino et al. 2018). A plethora of historical insect specimens are available from museum collections, including from important pollinator species. Further, natural history collections often extend more than a hundred years into the past (Holmes et al. 2016), thereby pre‐dating the major anthropogenic shifts of the 20th century (Steffen et al. 2015).

Insects from museum collections are usually preserved dry‐pinned at room temperature. While DNA degradates under these conditions, posing issues like fragmentation, post‐mortem DNA damage at read‐ends due to depurination and deamination, and contamination from exogenous sources (Raxworthy and Smith 2021), museum collections have now been proven a valuable resource for genomic studies. Museum genomics has the potential to contribute to unravelling the causes and consequences of insect declines by establishing historical baselines in genomic diversity and therefore help inform conservation practices (Nakahama 2021; Díez‐Del‐Molino et al. 2018). This potential has already been realized, for example revealing a decline in genomic diversity in Finnish butterfly species (Gauthier et al. 2020), pesticide adaptation and decline of genetic diversity in eastern honeybees in China (Liu, Qiu, et al. 2025), low genetic diversity without further decline prior to the putative extinction of *Bombus franklini* (Schweizer et al. 2025), and the effect of an invasive parasite on genetic diversity in wild honeybees in the USA (Mikheyev et al. 2015).

Bumblebees (*Bombus* spp.) are among the most important pollinators because of their ability to buzz‐pollinate, their ability to fly under cold conditions, and their eusociality (Goulson 2009; Velthuis and van Dorn 2006; Rasmont et al. 2015; Orlova 2025). Unfortunately, bumblebees have been declining in abundance since the 1950s (Ghisbain et al. 2025). These declines are largely driven by agricultural intensification, including, for example, the loss of nest sites, pesticide use, the introduction of non‐native bees and their pathogens, and reduced flower availability (Williams and Osborne 2009; Goulson et al. 2008). Since bumblebees are cold‐adapted, global warming is also an important driver projected to have caused strong and widespread declines (Soroye et al. 2020) and will likely result in further range contractions in the future (Ghisbain et al. 2024). The interaction of the different factors resulted in more than 25% of European bumblebee species, in particular specialists, being threatened with extinction (Ghisbain et al. 2025; Kosior et al. 2007; Dupont et al. 2011).

The common carder bee, *Bombus pascuorum* (Scopoli 1763), is a common and widespread bumblebee species in Europe, represented by 24 subspecies (Lecocq et al. 2015), and currently listed as Least Concern (Rasmont et al. 2015). With large populations (Liu et al. 2024) and the ability to link patchy plant populations (Feigs et al. 2022), this species is important for pollination. *B. pascuorum* seems to be increasing in abundance in Denmark (Kosior et al. 2007), Belgium, and Estonia (Maebe et al. 2015), and has remained stable in the UK (Casey et al. 2015). In Sweden, a decline in relative abundance has been documented in red clover fields using historical records (Bommarco et al. 2012) and, on a wider scale, using citizen‐science data combined with museum samples (Blasi et al. 2023). Unfortunately, the accuracy of studies relying on historical abundance data is only relative. For example, the increase of one species, such as *B. terrestris* , could reduce the relative abundance of *B. pascuorum* even if their populations have not changed. Therefore, alternative methods are required to fully understand the temporal population trend of this species.

In this study, we generate a reference genome for an individual of *B. pascuorum* collected in northern Sweden. We sequence a large number of genomes from museum specimens collected throughout Sweden during the last 150 years and analyse them together with a panel of diverse modern genomes. The main aim is to investigate geographical and temporal changes in genomic diversity and structure as a consequence of the putative population declines.

## 2 Materials and Methods

### 2.1 Sampling and Dataset

**FIGURE 1:**
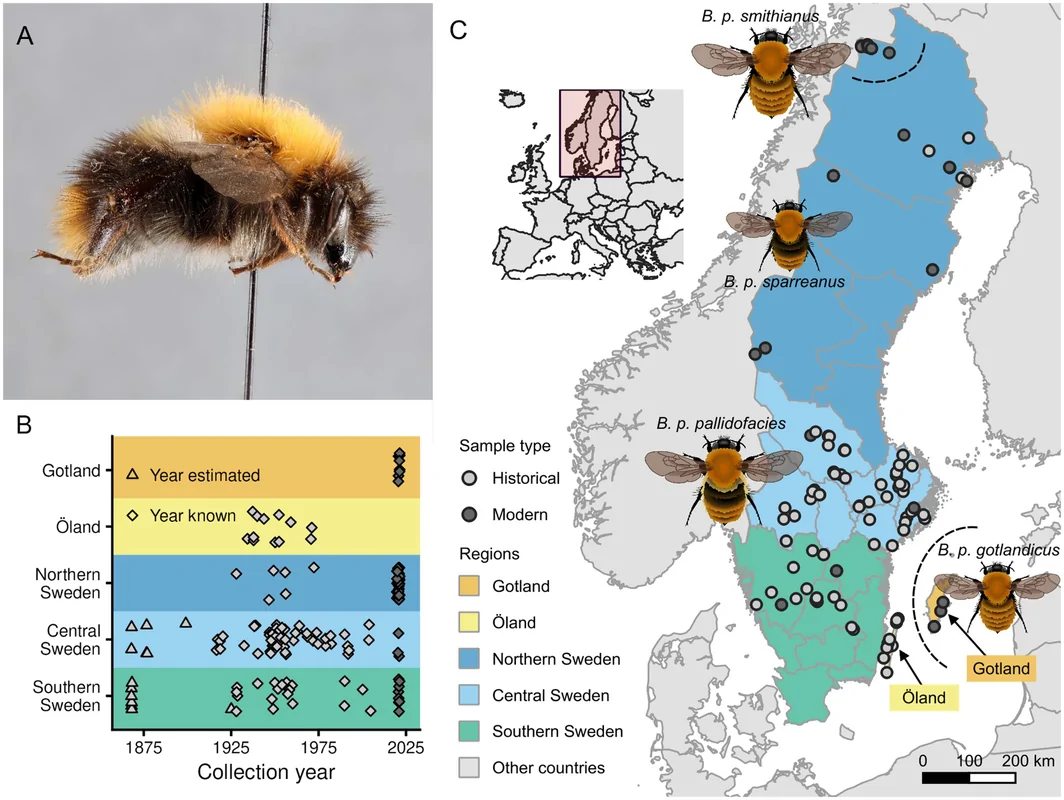
Spatial and temporal distribution of samples in this study. (A) Example of a dry‐pinned specimen of the study species, *Bombus pascuorum*, from the collections of the Swedish Museum of Natural History. (B) Collection date for the samples analysed in this study. Samples are coloured according to tissue type either as orange for historical dry‐pinned specimens or as dark blue for modern fresh tissue. Historical samples without an exact collection date are shown as a triangle. (C) Geographical location of the historical and modern samples in this study. There are samples of four subspecies in this study. *B. p. gotlandicus* is found on the island of Gotland and *B. p. smithianus* is found in the arctic mountains. *B. p. sparreanus* occurs in Northern Sweden and *B. p. pallidofacies* in Southern and Central Sweden, but the transition between these two is seamless.

We retrieved a total of 102 historical specimens of *Bombus pascuorum* from the Hymenoptera collection of the Museum of Natural History, Stockholm, Sweden (NHRS). For specimens missing a collecting date, we established the year of death of the collector as a conservative age for the sample. Modern bumblebees (*n* = 6) were collected in 2020 by sweep‐netting and then identified morphologically as *B. pascuorum* . These samples were stored in 80% ethanol at −20°C until laboratory analysis. One of these specimens, a female collected in Boden (Norrbotten, Sweden) in 2020 (Nb2020A), was used to generate the reference genome. Finally, our dataset was complemented with 53 previously published genomes (PRJNA890771) of Swedish *B. pascuorum* collected in 2021 (Liu et al. 2024). Therefore, the final dataset for this study was composed of genomes from 161 samples, 102 historical and 59 modern (Figure 1). The complete collection of metadata from both newly sequenced and previously published samples used in this study can be found in Table S1.

### 2.2 Laboratory Procedures

We generated a reference genome for Swedish *Bombus pascuorum* following the method described by Podsiadlowski et al. (2021). First, high molecular weight DNA was extracted from the thorax of a female specimen using the Nanobind Tissue Big extraction kit (Circulomics Inc.). The ethanol the specimen was preserved in was removed by washing and then rehydrating the tissue in an ethanol removal buffer (400 mM NaCl, 20 mM Tris pH 7.5, 30 mM EDTA). We then submerged the tissue in liquid nitrogen and ground it using a ceramic pestle. Thereafter, we followed the Circulomics protocol for the DNA extraction. To ensure a higher proportion of long reads, DNA fragments smaller than 10 kb were removed using the Short Read Eliminator Kit XS (Circulomics). Final DNA concentration was estimated using Nanodrop (ThermoFisher, MA) and Qubit (ThermoFisher). We constructed Nanopore sequencing libraries using the LSK‐110 ligation kit (Oxford Nanopore Technologies, UK, modified after Circulomics). Briefly, NEB end‐prep and repair times were extended 6 to 30 min at 20°C and 30 min at 65°C, adapter ligation time was extended to 1 h, and the elution of magnetic beads was extended to 20–60 min. All long‐read data were generated using a single MinION R9.4.1 flow cell in the modern DNA laboratory at the Centre for Palaeogenetics, Stockholm.

We generated Illumina short‐read data from the 6 modern *B. pascuorum* samples collected in 2020. A Blood and Tissue (Qiagen) kit was used to extract DNA from a piece of the thorax muscle. TruSeq PCR‐free libraries of 350 bp average insert size were generated following Illumina standard protocols and then sequenced on an Illumina NovaSeq S4 flowcell using a 2 × 150 bp paired‐end setting at NGI Stockholm.

In order to avoid contamination from modern DNA, we processed all historical samples in the designated historical DNA laboratories at the Centre for Palaeogenetics, Stockholm. A leg, preferentially the right meso leg, was removed from the specimens with sterile tweezers. Using a non‐unique structure for DNA extraction minimizes the damage to the specimen (Raxworthy and Smith 2021). To extract DNA from the legs, we adapted Protocol C (Brace et al. 2012), which is optimized for extracting short and very short DNA fragments. Briefly, the leg of each specimen was submerged in a lysis buffer (1 M Tris–HCl, 5 M NaCl, 1 M CaCl2, 0.5 M EDTA, 10% SDS, DTT) designed for chitin‐rich tissues (Campos and Gilbert 2019; Gilbert et al. 2007) together with 1000ug of proteinase K for 4 h, then, to release all possible DNA in the tissue, the leg was crushed using sterilized plastic pins and incubated overnight. The supernatant was concentrated using Vivaspin columns (Cytiva) and cleaned up using a MinElute PCR Purification Kit (Qiagen). We built dual‐indexed libraries according to Meyer and Kircher (2010). DNA extracts were first treated with USER enzyme (New England Biolabs) to remove deaminated bases caused by post‐mortem damage (Briggs et al. 2010). The extracts were then blunt‐end repaired, adapter ligated, and filled in following the protocol. We amplified and indexed the libraries with PCR cycles ranging from 8 to 14, depending on qPCR results. Finally, we used AMPure magnetic beads (Beckman Coulter Inc.) to remove long fragments potentially arising from contamination with modern DNA, as well as residuals with too small fragment sizes (i.e., adapters). The libraries were sequenced using paired‐end 2 × 150bp set up on an Illumina NovaSeq S4 or NovaSeq X Plus at NGI Stockholm.

### 2.3 Bioinformatics

For the reference genome, base calling from the raw ONT sequencing data were performed using Guppy v4.2.2 (Oxford Nanopore Technologies). The assembly was done using the Shasta long‐read assembler v0.7 (Shafin et al. 2020), a modified version of the NanoporeSep2021 config. The genome size was estimated to be ~250 Mb. The resulting draft assemblies were then polished with the same ONT sequence data used for the assembly with the pepper‐polish pipeline v0.1 (https://github.com/kishwarshafin/pepper), to improve base accuracy and reduce assembly errors. The assembly was additionally polished with Illumina short‐read data generated from the same specimen with POLCA (from MaSuRCA v.4.0.2) (Zimin and Salzberg 2020). We created a haploid assembly by filtering for alternative haplotypes using purge_dups v.1.2.5 (https://github.com/dfguan/purge_dups). The final polished genomes were assessed for repetitive content using RepeatModeler and RepeatMasker (Flynn et al. 2020). The quality of the newly assembled reference genome was assessed using QUAST (Gurevich et al. 2013) and BUSCO v.3.0.2 (Simão et al. 2015) with the ‘hymenoptera_odb10’ data set (*n* = 5991 genes).

We mapped all sequencing data to the generated reference genome using GenErode v0.5.1 (Kutschera et al. 2022), which processes historical and modern DNA jointly to make them comparable. For historical samples, adapters were trimmed, and paired‐end reads were merged with fastp v0.22.0 (Chen et al. 2018) using a minimum overlap of 15 bases, a maximum number of 3 mismatches in the overlapped region, and a percentual difference limit of 7%. GenErode mapped the data to the reference genome using BWA aln v0.7.17‐3‐deb_cv1 (Li and Durbin 2009) with ancient DNA settings (−l 16,500 ‐n 0.01 ‐o 2), sorted, indexed, and realigned mapping reads around indels, as well as removed duplicates using both start and end coordinates. Given the extremely short read lengths in historical insect samples (Mullin et al. 2023), we initially kept mapped reads of length > =20 bp as well as with a mapping quality of > = 25. Then, we used AMBER (Dolenz et al. 2024) with default settings to identify the optimal minimum read length cutoff that would allow us to keep as much data as possible without introducing an excess of mismatches. This is because, in very short reads, mismatch rates relative to the reference genome are typically higher due to bacterial‐derived spurious mappings (Dolenz et al. 2024).

For modern samples, mapping to the reference genome was done using BWA mem v.0.7.17 (Li 2013) with default settings. Mapped reads were sorted, indexed, and realigned around indels. Finally, any possible duplicates were marked using picard MarkDuplicates https://paperpile.com/c/yLMoOb/eMJWY (Picard Toolkit 2019) with default settings and removed with SAMtools v1.9 (Danecek et al. 2021).

All bam files were filtered by removing repetitive regions from the bam files by removing all reads overlapping these regions using BEDTools v1.20 (Quinlan and Hall 2010) using a repeat‐masked BED file created in GenErode using RepeatMasker and RepeatModeler v2.0.4 (Smit and Hubley 2008–2015). Finally, we established an additional filter that required all reads to have a minimum of 90% matching to the reference genome (Skoglund et al. 2012) for historical samples. For modern samples, this identity filter threshold was set to 95% to compensate for the longer read lengths. Mapping and sequencing statistics were obtained from qualimap v2.2.2 (Okonechnikov et al. 2016) and SAMtools v1.9 (Danecek et al. 2021). The change in read length was estimated with a segmented linear regression using the package segmented (Muggeo 2008) in R Studio.

### 2.4 Computational Analyses

Bumblebees have a haplodiploid sex‐determination system, where males are typically haploid and females diploid. Since both sexes are present in our dataset, we performed (pseudo)haploid variant calling in all individuals using ANGSD v0.940 (Korneliussen et al. 2014) by sampling a random base (‐‐dohaplocall 1) to avoid potential biases introduced by varying ploidy. We only allowed bases with a minimum base quality of 30 and a minimum mapping quality of 25. Mapping quality was adjusted for excessive mismatches (‐C 50), BAQ computation was performed (‐baq 1), bad reads were removed (‐‐remove_bads 1), and only uniquely mapping reads were included (‐‐uniqueOnly 1). Finally, we only retained biallelic sites and filtered out sites with a minimum minor allele frequency of < 5% or missing in more than 5% of the samples using PLINK v1.9 (Purcell et al. 2007). To avoid potential biases in all downstream analyses, we identified potential relatedness across all samples, historical and modern, using READ (Monroy Kuhn et al. 2018). After removing two related samples, our final dataset included all remaining 161 samples and 107,099 SNPs for population genomic analyses.

To investigate temporal changes and spatial patterns in population structure, we used Principal Component Analyses (PCA) in PLINK. We further investigated population structure using ADMIXTURE v1.3.0 (Alexander et al. 2009). We ran 10 replicates for each *k* = 1 to *k* = 10 with the haploid setting enabled (‐‐haploid = ‘*’) and a random seed set (‐‐seed) for every run.

To investigate the contribution of isolation‐by‐distance (IBD) to population structure, we calculated pairwise genetic distance by taking the Euclidean distance between the coordinates of the PCA axes (Shirk et al. 2017). We calculated the pairwise Euclidean distance between all 20 axes. We accounted for the differences in axis support by weighting the Euclidean distance with the explained variance of each axis. Geographical distances between locations were estimated from the collection coordinates using the geopy v2.4.1 package (GeoPy Contributors 2006) in Python v3.11.6. Only samples for which geographical information on the collection site was available were included in the IBD analyses. To investigate whether time played a role in genetic differentiation, we estimated isolation‐by‐time (IBT) by comparing the pairwise genetic differences calculated above against the differences in age between the samples. Specimens with no specific collection date were not included in these analyses. We excluded samples from the Arctic mountains and Gotland (Figure 1C), since we did not have historical samples from these areas (129 samples, 85,339 SNPs). For all isolation analyses, Mantel tests between distance matrices were calculated with 9999 permutations using the vegan package (Dixon 2003) in R v4.1.2 in RStudio (v2023.09.1, build 494). Additionally, we used a multiple regression model with the ecodist package (Goslee and Urban 2007) to investigate potentially conjoined effects of spatial and temporal distance.

We further investigated temporal and spatial differentiation in our dataset using weighted F_ST_ with VCFtools v0.1.16 (Danecek et al. 2011) using default settings. We ran spatial F_ST_ analyses by grouping samples based on the geographic regions of mainland Sweden and its islands. Samples from the arctic mountains identified as *B. p. smithianus* were treated separately from the rest of northern Sweden due to their distinctiveness in morphology, as well as their distinct clustering in PCA and Admixture (number of individuals: Southern Sweden: 34, Central Sweden: 61, Northern Sweden: 22, Arctic mountains: 24, Öland: 12, Gotland: 8). Temporal F_ST_ analyses were run by grouping samples in decades depending on their collection date. Samples older than 1900 were merged into one group. Modern samples not belonging to *B. p. sparreanus* or *B. p. pallidofacies* were excluded from the temporal F_ST_ analyses (number of individuals: pre 1900: 12, 1910–1919: 4, 1920–1930: 7, 1930–1939: 8, 1940–1949: 17, 1950–1959:26, 1960–1969: 8, 1970–1979: 8, 1990–1999: 6, 2000–2009: 4, modern: 27).

Since male bumblebees are typically haploid and female bumblebees are diploid, we used genome‐wide heterozygosity estimates to identify the genetic sex of all individuals using mlRho v2.9 (Haubold et al. 2010). For each sample, we prepared the input files using samtools mpileup and filtered out reads with a mapping quality < 25 and a base quality of < 30. Then, we filtered sites for coverage, including sites with a minimum depth of 3× and a maximum depth of 100×. The threshold for sex identification is 0.5 heterozygous sites per 1000 bp. Higher heterozygosity is considered diploid and therefore female; lower heterozygosity is considered haploid and therefore male.

To investigate temporal changes in individual genome‐wide heterozygosity, measured as theta (*θ*), we used mlRho on each diploid female. In order to include as many individuals as possible in this analysis, we first downsampled all female samples to an average genome depth of 2× and excluded females with lower depth in order to avoid biases caused by differences in sequencing depth. Then, within each individual, we restricted the mlRho analyses to sites with a sequencing depth of at least 3× in order to increase the likelihood of detecting at least one alternative allele for heterozygous sites. We also excluded any sites with a depth over 100× to avoid any remaining extremely high depth sites caused by spurious mappings. After downsampling to an average coverage of 2×, 62.8–69.6 M sites (average: 68.8 M) per sample remained at 3× or higher. We then performed the analyses with mlRho on the remaining 41 samples applying the same MQ and BQ filters as above.

Finally, we estimated relative nucleotide diversity (𝛑) using pixy v1.2.5 (Korunes and Samuk 2021) to test our filtered dataset for temporal changes in genetic diversity. Since nucleotide diversity is calculated pairwise, these analyses can be performed in the haploid SNP data of both diploid females and haploid males of all ranges of coverage. We ran nucleotide diversity analyses on the filtered variant file for the same sample groups described for the temporal F_ST_ analyses (161 samples and 107,099 SNPs). We used a window size of 10,000 sites and required windows to have at least one thousand SNPs. In order to avoid bias introduced by sample size differences, we calculated relative nucleotide diversity in groups of four samples for the temporal analyses, testing all possible permutations of samples within each population, and in groups of 8 samples for each group of samples, to a maximum of 1000 random combinations for the spatial analysis. We performed a linear regression on the mean nucleotide diversity of each decade in order to estimate the general temporal trend.

## 3 Results

### 3.1 Genome Assembly

We generated a *de novo* reference genome for Swedish *B. pascuorum* (*iyBomPascSwed.SU.1.0*). The final assembly had a length of 256.6 Mb and was composed of 128 contigs. The scaffold N50 was 8.7 Mb with 95% of the genome in scaffolds > 500 kbp long (Figure S1). BUSCO analyses showed that the assembly is highly complete with 5780 (96.5%) single‐copy genes, 15 (0.3%) duplicated, 54 (0.9%) fragmented, and 157 (2.6%) missing orthologs of the 5991 in the hymenoptera database (Figure S1). Repeatmasker analyses identified 48,9 Mbp (19,058%) of the reference genome as repetitive regions. The majority of these regions were annotated as unclassified interspersed repeats (8.37%), with other repeats such as LINEs (2.04%), LRT elements (2.52%), DNA transposons (3.22%), and simple repeats (2.43%) less represented (Table S2).

### 3.2 Historical DNA Data Characterization

**FIGURE 2:**
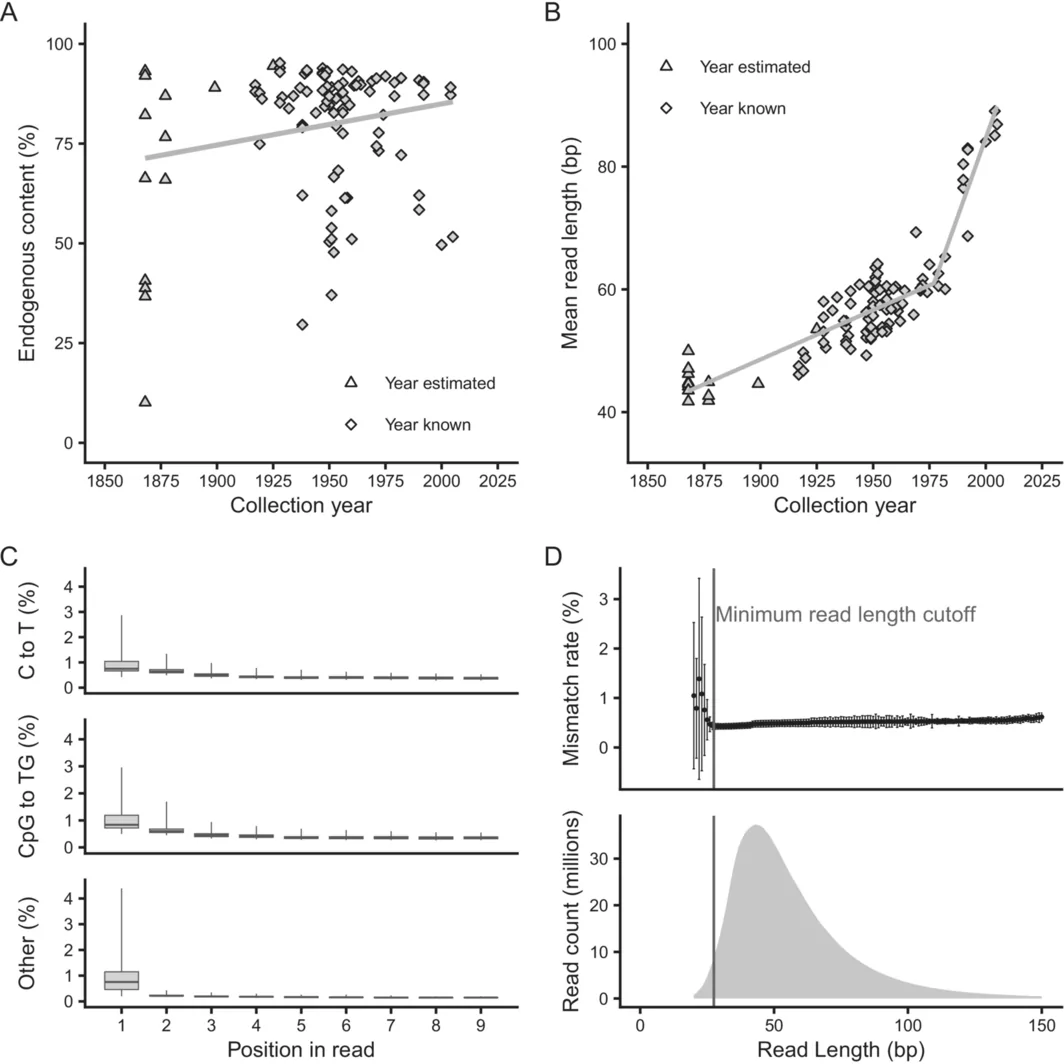
Characterization of historical bumblebee DNA. (A) Endogenous content of each sample, calculated as the proportion of raw reads mapping to the reference genome. Historical samples without an exact collection date are shown as a triangle, a trend line is given in grey (linear model: Adjusted R‐squared: 0.03, *p*‐value: 0.046). (B) Per sample mean merged read length (bp) of the retained reads after filtering compared to the specimen's collection year. A segmented linear regression is shown as grey line, suggesting 1977 as breaking‐point (95% CI: 1972.5–1980, *p*‐value: < 2e‐16, Adjusted R‐squared; 0.866; slope before 1977: 0.016, slope after 1977: 1.03). (C) Post‐mortem damage at read ends shown for the first 9 bases of historical samples. (D) The mean and standard deviation of the mismatch rate and sum of read counts across all historical samples plotted against the read length. Vertical lines indicate the minimum read length cutoff (28 bp).

We successfully retrieved DNA for 102 historical specimens of *B. pascuorum* (Figure 1A), some of which date back more than 150 years. The endogenous content in our samples, estimated as the percentage of raw sequencing reads mapping to the reference genome, was high (median 86.9%, range 10.1–95.2). The endogenous content remained relatively stable with increasing sample age, although some samples exhibited lower endogenous content, particularly among the oldest ones (Figure 2A). For example, our lowest endogenous sample (Sm1868E) corresponded to a specimen collected by Carl Henrik Boheman sometime before 1868. After filtering, the final genomes had a median depth of 3× (range 0.13–18.8).

Our historical samples displayed very short merged read lengths (RL, Figure 2B) with an average of just 58.3 bp across all samples (range 41.8–89 bp). We further observed a declining trend in the per‐sample average RL that correlated with sample age, with older samples displaying more fragmented sequences than the younger ones. Segmented linear regression (Adjusted R‐squared: 0.866) revealed a pronounced RL declining rate of 1.03 bp per year (95% CI: 0.811–1.249) for samples collected in the early 2000s to about 1977 (95% CI: 1972.5–1980, *p* < 2e‐16). Before 1977, the decline in RL was at about 0.161 bp per year (95% CI: 0.135–0.187).

As expected after full USER treatment, we found only low levels of post‐mortem damage remaining at the read ends of our historical samples. This damage was most pronounced in the first base with a mean of 0.96% for C to T transitions, 1.08% CpG to TG sites, and 0.96% for other damage (Figure 2C). We did not find a correlation between the remaining post‐mortem damage at the first position and sample age (Figure S2).

AMBER analyses indicated pronounced peaks in mismatch rates at very short read lengths (< 25 bp) in the historical samples (Figure 2D, Figure S7), probably corresponding to contaminant sequences. However, the fragment size distribution across all historical samples showed that there were > 21 M sequences with mismatches comparable to longer RLs below the read length of 30 bp, the most common RL cutoff in ancient DNA. To maximize the number of reads to keep for analysis, we therefore aimed at identifying the minimum read length possible without increasing the mismatch rate. We estimated the mean mismatch rate for all samples at each read length as well as the standard deviation (SD). Since the mean mismatch and SD were high in very short reads, but became constant at 28 bp, we set the minimum read length cutoff of all samples to 28 bp (Figure 2D). Compared to using 30 bp as the default threshold, using this method, we increased the number of retained reads per sample by a mean of 210,683 (13,072–930,347), an average of 1.5% of the total per sample reads (0.09%–7.22%).

### 3.3 Population Structure

We did not find evidence of relatedness between any of the historical bumblebee samples in this study. However, the kinship results indicated that two modern bumblebees were identical twins (DA_02 and DA_03). Since these were collected in two different locations more than 200 km apart, we excluded both samples from all downstream analyses. After all filtering, we retained 107,099 SNPs for population genomic analysis.

**FIGURE 3:**
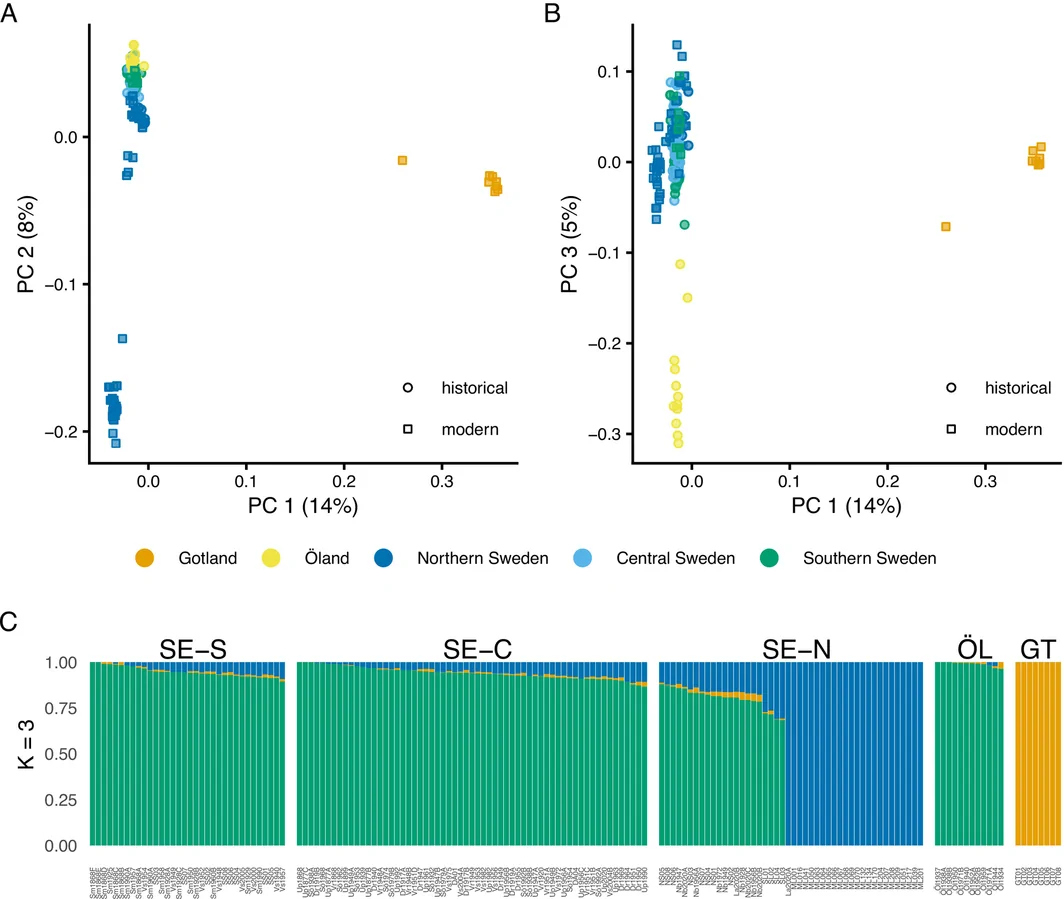
Spatial population structure of *Bombus pascuorum* in Sweden. (A) The first two axes of a Principal Component Analysis including all samples in this study, coloured by region. (B) The third and the second axis of the PCA including all samples. (C) The admixture analysis for *K* = 3. Fill is chosen to fit the regional patterns from the PCA. Samples are separated by region (SE‐S: Southern Sweden, SE‐C: Central Sweden, SE‐N: Northern Sweden, Öl: Öland, GT: Gotland). Samples are sorted by admixture proportion within each region.

We first investigated geographical population structure and putative changes to it during historical times using Principal Component Analyses. The first axis separated the samples from the island of Gotland and samples from Sweden's arctic mountains were separated from the rest along the second axis (Figure 3A). Within the main cluster, we found samples from Northern Sweden slightly segregating from those from Central and Southern Sweden. This population structure generally matched the subspecies described based on colour patterns, with *B. p. gotlandicus* on Gotland, *B. p. smithianus* in the arctic mountains, *B. p. pallidofacies* in Southern and Central Sweden and *B. p. sparreanus* in Northern Sweden. Additionally, we found that individuals from the island of Öland, located close to the eastern coast of mainland Sweden, were separated along the third PC axis (Figure 3B).

We further analysed population structure using admixture analyses. Whereas the lowest CV‐value suggested *k* = 1 as the optimal model for our dataset (Figure S3), *k* = 3 indicated a similar population structure as found in the PCA (Figure 3C) with samples from Gotland and the arctic mountains separating from the rest. Also, in *k* = 4, Öland samples were separated from mainland Sweden (Figure S4).

### 3.4 Population Differentiation

**FIGURE 4:**
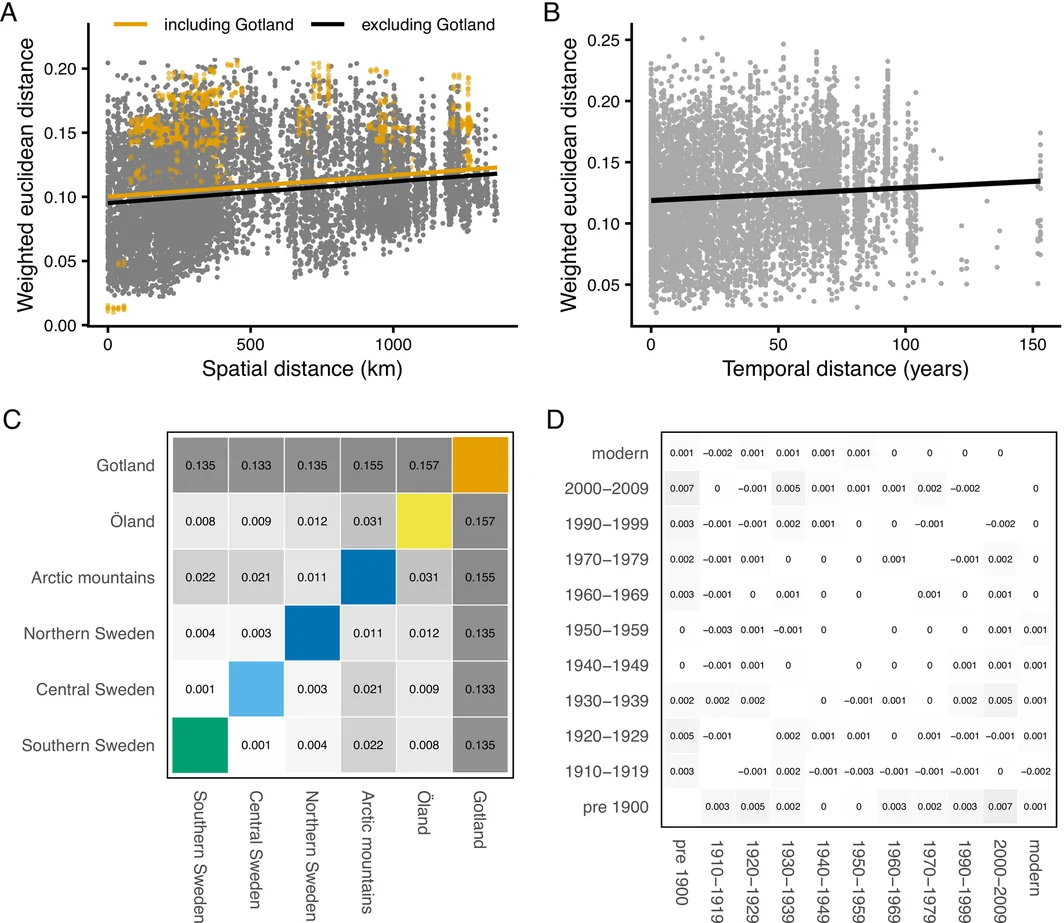
Population differentiation of *Bombus pascuorum* in Sweden. (A) Isolation‐by‐distance analyses between genetic distances and spatial distance for all samples. Since the sea likely acts as a barrier isolating the subspecies from Gotland, we highlighted combinations including samples from Gotland in orange. Comparisons are shown as dots and a linear regression is shown for visualization purposes only, in black excluding Gotland and in orange including all samples (Mantel statistic including Gotland: R: 0.1739, significance: 1e‐04, excluding Gotland: R: 0.1897, significance: 1e‐04). (B) Isolation‐by‐distance analyses between genetic distances and temporal distance, excluding individuals from *B. p. smithianus* and *B. p. gotlandicus*. Comparisons are shown as dots and a linear regression is shown in black for visualization purposes only (Mantel statistic r: 0.07552, significance: 0.0478). (C) F_ST_ for samples in the different regions. Samples from the arctic mountains were separated from the rest of Northern Sweden. Darker grey indicates a larger F_ST_ value. (D) F_ST_ between individuals collected in different decades, excluding samples from Gotland and the arctic mountains.

We used isolation‐by‐distance (IBD) analyses to investigate the genetic differentiation patterns that could explain the observed population structure in Swedish *B. pascuorum* . We first examined whether geographical distance explains the observed genetic distances, measured as the weighted Euclidean distance of the first 20 PCA axes between each pair of samples. Our results indicated that geographical distances explain a small but significant part of the observed genetic distances (Mantel statistic r: 0.1739, significance: 1e‐04; when excluding Gotland: r: 0.1897, significance: 1e‐04, Figure 4A). We also estimated isolation‐by‐time (IBT) by comparing the genetic distances to the difference in collection years between each pair of samples. To avoid biases in our temporal analyses, we excluded all samples from the arctic mountains and the island of Gotland from these analyses since we did not have any historical samples from those locations. Our results indicated that temporal distances explain an even smaller portion of the observed genetic distances (Mantel statistic r: 0.07552, significance: 0.0478, Figure 4B). The multiple regression model showed an overall low impact of both space and time on genetic distance (R^2^: 0.021, *p*‐value: 0.043, *p*‐value time: 0.237, *p*‐value geographical: 0.059). Finally, we examined more granular patterns of differentiation by investigating the IBD between samples grouped by decade. We observed a stronger IBD in the 20s, 30s, 60s and 70s than in modern samples, but the respective mantel tests were not significant (Figure S5).

We also investigated population differentiation using F_ST_ analyses (Figure 4C). We first tested geographical differentiation by grouping samples based on their geographical location as well as the PCA and admixture results. We found the strongest differentiation between the Gotland samples and all the other groups (0.133–0.157). The samples from the arctic Swedish mountains, *B. p. smithianus*, were the second most differentiated from all other groups (0.011–0.031). The F_ST_ value was low between samples from Öland and the mainland populations (F_ST_ = 0.008–0.012), and even lower across all samples from Northern, Central and Southern Sweden (F_ST_ = 0.001–0.004). We also estimated temporal F_ST_ values grouping samples by decade (Figure 4D). Again, all samples from the arctic mountains and the island of Gotland were excluded in order to avoid biases. Our results indicated very low temporal F_ST_ (0–0.007) in all comparisons.

### 3.5 Genomic Diversity

**FIGURE 5:**
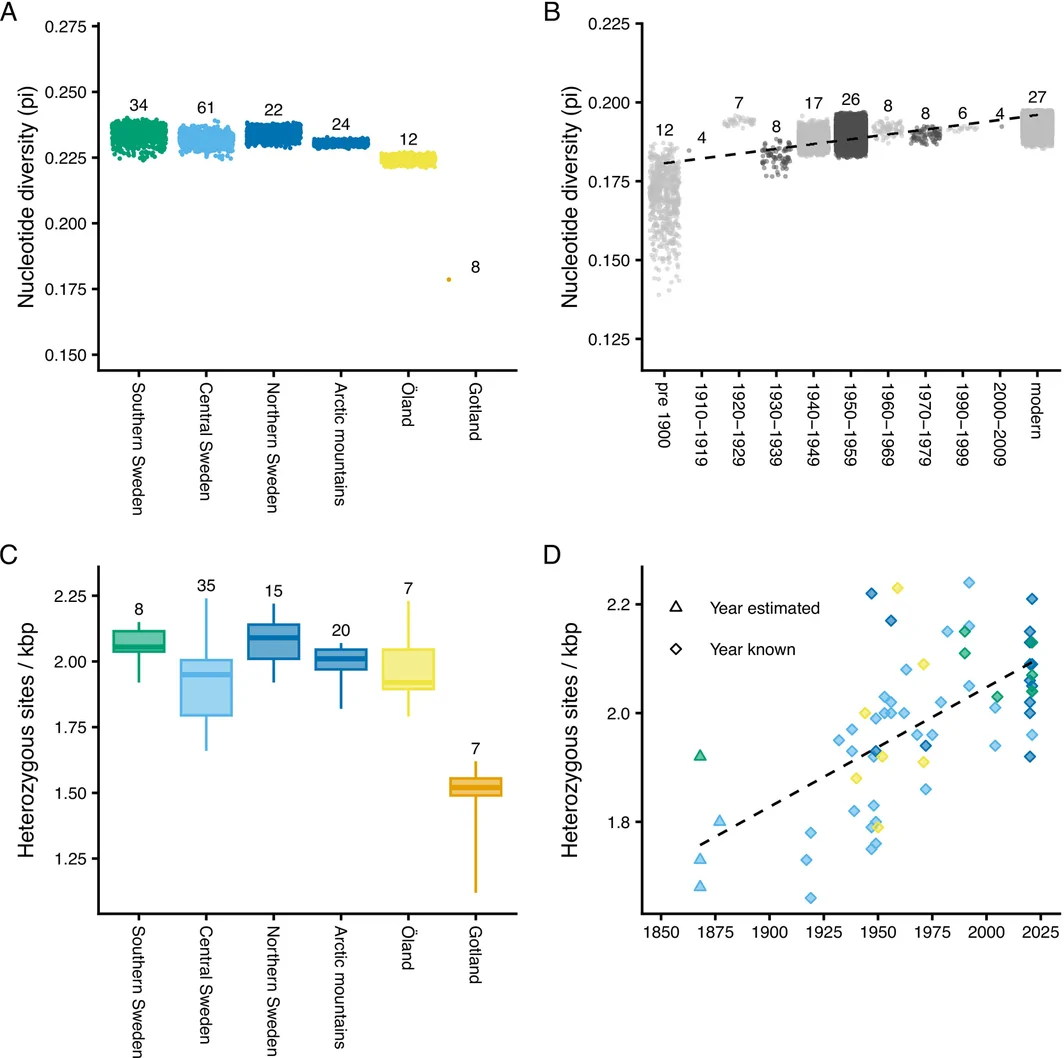
Genetic diversity in *Bombus pascuorum* in Sweden. (A) Nuclear diversity (Pi) for variant sites in samples from different regions. Samples from the arctic mountains were separated from the rest of Northern Sweden to account for their differences in population structure. To account for the variation in sample size, pi was calculated for up to 1000 random combinations of 8 samples for population. The total number of samples per population is given above each group. (B) Nucleotide diversity (𝛑) for variant sites across samples collected in different decades, *B. p. smithianus* and *B. p. gotlandicus* were excluded. Each dot represents a group of four individuals from within each population, all possible combinations of samples are shown. A linear regression was calculated for the means of the decade (adjusted *R* ^2^ = 0.426, *p* = 0.0176) and projected as a dashed line. (C) Heterozygosity of females compared between regions. Boxplots are formed by the results for the individuals within each population. (D) Estimated individual genome‐wide heterozygosity through time, individuals from Gotland and the arctic mountains were excluded. A dashed line is added for the linear model (Adjusted R‐squared: 0.4247, *p*‐value: 2.48e‐09, slope: 0.0023).

We used nucleotide diversity (𝛑) to characterize genomic diversity in Swedish *B. pascuorum* across time and space (Figure 5A,B). Genomic diversity was lowest in the Gotland (𝛑 ~0.179) and Öland islands (𝛑 ~0.230), with samples from the other locations having a relatively similar nucleotide diversity (𝛑 ~0.242–0.247, Figure 5A). In the analyses grouping samples by collection year and excluding individuals from *B. p. smithianus* and *B. p. gotlandicus*, we found that nucleotide diversity was slightly variable between decades (mean 𝛑 ~0.169–0.194, Figure 5B). While mean nucleotide diversity increased over time (by ~0.0012 per decade, adjusted *R* ^2^ = 0.426, *p* = 0.0176), all groups except for the permutations before 1900 were within a tight range of nucleotide diversity values (mean 𝛑 ~0.183–0.194, Figure 5B).

We used the fact that bumblebees have a haplo‐diploid system with haploid males and diploid females to perform genetic sex determination by estimating genome‐wide heterozygosity in each individual (Figure S6). This allowed us to identify genetic sex for a limited number of specimens lacking this information from the collection metadata, as well as to detect possible discrepancies between the morphological sex documented for the specimen and the genetic sex. We were particularly interested in the potential presence of diploid males, which are a sign of strong inbreeding in bumblebees (Cook and Crozier 1995). Finally, the genetic sex was used to identify diploid individuals for heterozygosity estimation and to check for a potential impact of heterozygosity on the population genomic analyses. We observed a clear gap between samples with a low number of heterozygous sites per kbp (< 0.17), which we described as genetically haploid (males), and samples with 1.38 or more heterozygous sites per kbp, which we described as genetically diploid (females). We did not find any differences between historical and modern tissues. However, there was one individual described as female with a very low number of heterozygote sites per kbp (0.0519 s/kbp) and one individual described as male with a high heterozygosity (2 s/kbp). A morphological re‐identification of the museum specimen of the potential diploid male confirmed its status as a female.

Finally, we used mlRho to estimate genome‐wide heterozygosity for each diploid female, measured as theta (*θ*). We first grouped the females according to their geographical origin (Figure 5C). Gotland had the lowest heterozygosity (avg. 1.48 sites/kbp) and Central Sweden had a slightly reduced heterozygosity (avg. 1.91 sites/kbp), while all other groups had similar heterozygosity (avg 1.97–2.07 sites/kbp). To investigate changes through time, we removed individuals from the Arctic mountains and the island of Gotland to avoid potential bias arising from the lack of historical samples from these areas. We observed a significant global increase in genomic diversity of ~0.06 heterozygous sites per kbp every 25 years (95% CI: 0.0017–0.0031 sites per year, *p*‐value: 2.48e‐09), a total of 0.36 heterozygous sites per kbp in the past 150 years (Figure 5D).

## 4 Discussion

### 4.1 A Reference Genome for Swedish *B. pascuorum*

In this study, we present a large‐scale genomic dataset of Swedish *B. pascuorum* spanning over wide spatial and temporal scales. Despite strong evidence for declines in abundance (Sánchez‐Bayo and Wyckhuys 2019; Wagner et al. 2021; Cardoso et al. 2020), studies on insect conservation genomics are still rare (Webster et al. 2023; Halsch et al. 2025), partially due to the absence of appropriate reference genomes (Sucháčková Bartoňová et al. 2023; Li et al. 2025). Fortunately, in recent years this limitation has been increasingly addressed (Hotaling et al. 2021), and now several international consortia (i.e., DToL, ERGA) are generating reference genomes for all living organisms, including insect pollinators such as bumblebees. In fact, DTOL recently generated a reference genome for *B. pascuorum* , from a specimen collected in the UK, where the subspecies *B. p. vulgo* occurs (Crowley et al. 2023). Given the high variability existing for this species in Europe, where up to 24 subspecies have been described (Lecocq et al. 2015), it is possible that this reference genome would not be able to fully capture local variation in divergent subspecies. We therefore used long‐read sequencing to successfully generate a high quality reference genome for *B. pascuorum* from a specimen collected in northern Sweden.

### 4.2 Historical DNA in Insect Conservation Genomics

A common limitation to conservation studies in insects is the lack of genomic data on a temporal scale, which has been highlighted as fundamental to assess, monitor and predict population declines (Nakahama 2021; Díez‐Del‐Molino et al. 2018). Unfortunately, there are technical difficulties associated with working in historical DNA, including contamination from modern DNA sources, DNA fragmentation, and DNA damage (Raxworthy and Smith 2021). In fact, so far there have only been a limited number of conservation genomics studies using insects from historical collections (see for example Gauthier et al. 2020; Whitla et al. 2024; Schweizer et al. 2025; Liu, Qiu, et al. 2025).

While a low endogenous content, defined as the proportion of sequences mapping to the reference genome, is common in ancient as well as in historical samples (von Seth et al. 2021; Larsson et al. 2019; Ferrari et al. 2023), our museum sampled bumblebees show an overall high proportion of endogenous sequences (avg. 79.3%). This is coherent with previous observations in whole genome sequencing of dry‐pinned insects. For example, Mullin et al. (2023) described endogenous contents of around 90% in historical bumblebees sequenced from various museum collections around the UK, and Whitla et al. (2024) obtained an average of 77% in pin‐dried butterfly specimens from museums in France and the UK. These results suggest that, despite being stored during decades and up to 150 years in unfavourable conditions for DNA preservation, which can vary wildly across museum collections (Mullin et al. 2023), dry‐pinned insect specimens can contain large amounts of DNA, rendering them usable for conservation research based on whole genome sequencing, such as this study.

One striking characteristic of our historical insect DNA is the high degree of fragmentation. We found an average merged read length as low as 42 bp for samples collected more than a hundred years ago. This level of fragmentation, roughly equivalent to that found in permafrost‐preserved million‐year‐old mammal samples (van der Valk et al. 2021), has been observed in all previous genomics studies in dry‐pinned insects (e.g., Shpak et al. 2023; Cavill et al. 2022; Whitla et al. 2024) and highlights the need for increased sequencing effort to recover the complete genomes of museum specimens despite their high endogenous content. While Mullin et al. (2023) described a linear relationship between fragmentation and collection date across several museums, we found an accelerated rate of DNA fragmentation (10.3 bp per decade until 1977) within the first decades after being dry‐pinned. Insect specimens that have only been stored for 15–20 years already have significantly shorter reads compared to libraries generated from fresh tissue. This matches the expectation that DNA fragmentation happens rapidly after the death of the insect, during the drying process (Sawyer et al. 2012). We also find that the rate of fragmentation greatly slows down for specimens collected more than fifty years ago (1.61 bp per decade), which enables us to obtain workable genomic data for samples far beyond that range.

Another consequence of having shorter sequences is that they are more likely to map incorrectly. In ancient and historical DNA the read‐length cutoff is usually set in the range of 30 to 35 bp (Kutschera et al. 2022) to avoid mapping errors in very short reads, which are usually caused by bacterial contamination (Dolenz et al. 2024). However, this is highly variable across samples and given the high levels of fragmentation, setting up standard, conservative read‐length thresholds can greatly reduce the amounts of data available for downstream analyses. In this study, we addressed this limitation by investigating the mismatch rates at short read lengths for each sample which allowed us to optimize a minimum read length cutoff that maximized the amount of data available up to 7% for some samples without introducing short sequences enriched with mismatches. We think this strategy has the potential to increase the amount of reliable sequencing data in other conservation genomics projects based on museum insect specimens suffering from high levels of DNA fragmentation.

### 4.3 Stable Population Structure and Genetic Diversity Over the Course of 150 Years

Despite the high morphological diversity described in *B. pascuorum* , our results suggest low global genetic population structure in Sweden. This is in line with current research on common bumblebees, where often very low (Glück et al. 2022; Cobb et al. 2024) or no population structure is found within extensive regions (Hart et al. 2022), while less common species such as *Bombus affinis* show stronger population structuring (Mola et al. 2024). A possible explanation is that genetic exchange among distant populations is facilitated by the bumblebee's high dispersal capacity. Although common foraging distances are usually just a few hundred metres (Redhead et al. 2016), there is evidence of long‐range migration in bumblebees (Fijen 2021), and *Bombus* species that are introduced to novel environments can travel many kilometres in a year (Kingston et al. 2002; Schmid‐Hempel et al. 2014).

While the global population structure of Swedish *B. pascuorum* is low, we do find the samples collected in Gotland and the Arctic Swedish Mountains genetically differentiated from the rest across our population structure and differentiation analyses. The most divergent sample group is on the island of Gotland, which have been described as the subspecies *B. p. gotlandicus*. This matches previous studies which described fine‐structure between bumblebee populations at short geographical distances as a consequence of ocean barriers reducing gene flow (Cobb et al. 2024; Jha 2015). In fact, we also find some genetic differentiation in the samples from the island of Öland compared to the mainland, despite the small distance to mainland Sweden, just 5 km in the narrowest point. Both the samples from Gotland and Öland exhibit lower heterozygosity and higher similarity than the others, potentially due to limited genetic exchange with the mainland populations and smaller population sizes, as suggested by the presence of higher levels of inbreeding in Gotland (Liu et al. 2024). The other genetically distinct cluster comprises all the samples from the arctic Swedish mountains, where the subspecies *B. p. smithianus* has been described. These samples display higher genetic differentiation with respect to all other mainland Sweden samples, despite being geographically closer to some of them. We therefore hypothesize that differentiation might be promoted by local adaptation of these bumblebees to more arctic/alpine habitats, but further research on the climatic barriers to gene flow in bumblebees is necessary. Interestingly, other bumblebee species have shown rapid adaptation to local environments despite limited population structure (Theodorou et al. 2018), highlighting their potential for local adaptation despite the existence of gene flow. The samples from the other two subspecies, *B. p. pallidofacies* and *B. p. sparreanus*, distributed over vast geographical distances from southern to northern Sweden, display no genetic substructuring within the mainland and only a very shallow isolation‐by‐distance differentiation pattern, both of which support a high degree of mobility for this species. These two subspecies are also much more difficult to identify as they likely form part of a south–north morphological continuum (Løken 1973). This is also consistent with genetic markers showing limited evidence for the existence of some of the morphologically described subspecies in *B. pascuorum* (Lecocq et al. 2015).

Anthropogenic activity can alter the population structure of species within short periods of time, for example, by adding barriers to dispersal, extirpating stepping‐stone populations, or increasing habitat fragmentation (Remon et al. 2022; Darvill et al. 2006). In this study, we used an extensive temporal dataset to investigate whether the observed patterns of population structure in Swedish *B. pascuorum* have been impacted by human‐induced changes to the landscape within the last 150 years. Our results indicate no differentiation in historical samples from across Sweden, and therefore suggest that *B. pascuorum* population genetic structure has not been significantly impacted by past anthropogenic activity.

Despite the proposed decline in relative abundance of *Bombus pascuorum* in Sweden (Bommarco et al. 2012; Blasi et al. 2023), we did not find a decline in genomic diversity through time. In fact, while nucleotide diversity varied slightly between decades, likely because of differences in the number of samples and their geographical origin, we found an overall increase in mean nucleotide diversity over time. Additionally, our results indicated a temporal increase in individual genome‐wide heterozygosity for females. This lack of a decline in genetic diversity fits the results from microsatellite data in Belgium, where stability in temporal genetic diversity was reported for several bumblebee species, including *B. pascuorum* (Maebe et al. 2016).

Several hypotheses could explain this observation. First, the large population sizes of Swedish *B. pascuorum* (Liu et al. 2024) relative to the magnitude of the abundance declines, coupled with the high dispersal capability of bumblebees (Fijen 2021), could allow this species to mitigate the effects of any putative population declines. Second, the population declines might be too recent to result in a detectable decline in genomic diversity (Webster et al. 2023). Even in a putatively extinct species, *B. franklini* , historical DNA data did not show a decrease in genomic diversity in the years prior to its disappearance (Schweizer et al. 2025). Other studies found a decline in genomic diversity, for example, over a period of more than a hundred years in the black‐veined butterfly, *Aporia crataegi* (Whitla et al. 2024), and in a period of about 60 years in the grasshopper *Oedaleus decorus* (Schmid et al. 2018). Nevertheless, a time‐lag between population decline and noticeable changes of genomic diversity has been reported on multiple other species (Manthey et al. 2025; Liu, Milesi, et al. 2025; Gargiulo et al. 2025) and could explain the observed patterns for Swedish *B. pascuorum*. Finally, *B. pascuorum* is currently one of the most common bumblebee species in central Europe (Kosior et al. 2007; Liu et al. 2024; Rasmont et al. 2015). Our findings could also suggest that the abundance declines are not major enough to cause any noticeable genomic erosion, that the reported temporal changes in relative abundance might not translate effectively to population declines, and there has not been a significant population decline in *B. pascuorum* in Sweden, or that the Swedish populations have been, in fact, in expansion since the beginning of last century. In fact, despite being regarded as a specialist species, having a medium to long proboscis (Løken 1973), *B. pascuorum* has been reported to increase in abundance in Denmark and Great Britain, profiting from declines of other long‐tongued species (Dupont et al. 2011; Plowright et al. 1997).

In conclusion, our study highlights the importance of historical genomic data to detect and assess past demographic declines and genetic erosion in insect pollinators. Implementing long‐term genomic monitoring programs can be crucial for tracking real‐time evolutionary dynamics, enabling conservationists to assess shifts in population size, detect subtle genomic diversity trends, and identify signs of decline long before they lead to irreversible genetic consequences. Conserving and restoring habitats, especially those in regions where genetic reduced diversity has been observed and local adaptation might be important, such as Gotland and the Arctic mountains, could be instrumental to avoid ongoing and future abundance declines in Swedish *Bombus pascuorum* before its populations show any genetic consequences.

## Author Contributions

David Díez‐del‐Molino conceptualized the project. Jovanka Suess collected modern samples and Hege Vårdal provided historical samples and metadata from the collection of the Naturhistoriska Riksmuseet (NHRS). Matthew T. Webster provided sequencing data and metadata from modern samples. David Díez‐del‐Molino, Ellen Karlsson, Jovanka Suess and Michael Mitschke performed laboratory work. Kalle Tunström, David Díez‐del‐Molino and Christopher W. Wheat generated the reference genome, and Ibai Ugarte‐Zabaleta performed quality control. Michael Mitschke, and David Díez‐del‐Molino analysed and interpreted the data. Michael Mitschke drafted the manuscript, figures, and illustrations. All authors contributed to writing, reviewing and editing the final manuscript.

## Funding

DDdM acknowledges support from the Carl Tryggers Foundation (17:109), VR (2023‐04467), and FORMAS (2022‐00379). CWW acknowledges support from the Swedish Environmental Protection Agency (Naturvårdsverket), who commissioned and funded the genome reference sequencing, especially Kristin Löwenborg for her participation in the work of formulating the project/commission, as well as support from the Swedish Research Council (2017‐04386). The authors thank the National Genomics Infrastructure (NGI) for providing assistance with sequencing. The computations and data handling were enabled by resources in projects (NAISS 2025/5‐78, NAISS 2025/22‐1510 and NAISS 2025/2‐10) provided by the National Academic Infrastructure for Supercomputing in Sweden (NAISS), funded by the Swedish Research Council.

## Conflicts of Interest

The authors declare no conflicts of interest.

## Supporting information


**Figure S1:** Assembly statistics and BUSCO results. (A) Cumulative genome size for each scaffold. 95% of the assembly is allocated in 54 scaffolds longer than 500 kbp. (B) Scaffold sizes in Mb. (C) BUSCO results using the *hymenoptera_odb10* database.
**Figure S2:** Average post‐mortem damage in the first base for each sample. (A) C to T, (B) CpG to TG, and (C) other damage are shown in comparison with sample age. Samples with a known age are shown as diamonds. Samples with an estimated year are shown as triangles.
**Figure S3:** Cross‐validation (CV) results for ADMIXTURE. The ten CV values for each K are shown as boxplots. The means across replicates for each K are connected by a line.
**Figure S4:** ADMIXTURE results for *K* = 2–4. Each vertical bar corresponds to a sample. Samples are separated by region and then sorted by admixture proportions in *K* = 3.
**Figure S5:** Isolation‐by‐distance results for each decade. Comparisons are shown as dots, and a linear regression is shown in black. Mantel test results are given in the respective panel. The samples from Gotland and the Arctic mountains are excluded.
**Figure S6:** Genetic sex determination using mlrho. Since bumblebees are haplodiploid, we used the heterozygosity estimates (theta) to determine genetic sex. For each sample, we compare the number of heterozygous sites per kbp with sequencing depth. Historical samples are coloured in light grey, modern samples in dark grey. We observe a clear gap between ~0.17 and ~1.38 heterozygous sites per 1000 bp, which we hence describe as the gap between genetic males and females. Specimens morphologically identified as males are shown as squares, females as circles, and those with missing data as rhombuses.
**Figure S7:** AMBER results for all historical samples. In each plot, the mismatch rate is shown in black dots against the read length. The values are described on the left y‐axis. The grey distribution curve indicates the number of reads in thousands, as shown by the right y‐axis. The read length cutoff is shown by the dark red vertical line.

**Table S1:** Metadata. This table contains the metadata for the samples used in this study. Each sample has three IDs, ‘ID’ is used in the text and figures, ‘Lab ID’ was used in the laboratory and bioinformatic procedures, and finally the museums or field ID. ‘Sample’ and ‘Tissue’ describe the sampling material, being either ‘historical’ and ‘dry‐pinned’, or ‘modern’ and ‘fresh’. The ‘Sex, morphological’ was retrieved from the labels on the historical samples and the ‘Sex, ploidy’ is the genetic sex identified by investigating ploidy, with haploid individuals as ‘Male’ and diploid individuals as ‘Female’. Latitude and longitude were not given for historical samples and were estimated based on the locality name. The ‘Year’ is derived from the ‘Date’ and for individuals with missing information it was extimated as the year of death of the collector as a conservative estimate of sample age. The assignment for population‐based analyses like Pi and Fst is given for the spatial (grouping for pi and fst, region) as well as the temporal analysis (grouping for pi and fst, decades). The samples were either sequenced for this study or retrieved from Liu et al. 2024, as shown in ‘Reference’.
**Table S2:** Results from RepeatMasker. In this table, we present the full report of the RepeatMasker for iyBomPascSwed.SU.1.0.

## Data Availability

The raw sequencing data and the reference genome generated in this study are accessible in ENA, project PRJEB115033. We also used publicly available data from project PRJNA890771 (Liu et al. 2024). The code is available on GitHub: https://github.com/MitschkeMi/Museum‐genomics‐of‐an‐important‐Swedish‐pollinator‐reveals‐population‐stability‐across‐time.
