# Supplementary material for: Museum Genomics of an Important Swedish Pollinator Reveals Population Stability Across Time
Source: Mol Ecol. 2026 Sep 22;35(18):e70557. doi: 10.1111/mec.70557 (PMC13598348; doi:10.1111/mec.70557)
Supplement: Supplementary file 1 [file MEC-35-e70557-s001.docx]

### Supplementary figures

#### Figure S1: Assembly statistics and BUSCO results. A) Cumulative genome size for each scaffold. 95% of the assembly is allocated in 54 scaffolds longer than 500 kbp. B) Scaffold sizes in Mb. C) BUSCO results using the *hymenoptera_odb10* database.

#### Figure S2: Average post-mortem damage in the first base for each sample. A) C to T, B) CpG to TG, and C) other damage are shown in comparison with sample age. Samples with a known age are shown as diamonds. Samples with an estimated year are shown as triangles.

#### Figure S3: Cross-validation (CV) results for ADMIXTURE. The ten CV values for each K are shown as boxplots. The means across replicates for each K are connected by a line.

#### Figure S4: ADMIXTURE results for K=2 - 4. Each vertical bar corresponds to a sample. Samples are separated by region and then sorted by admixture proportions in K=3.

#### Figure S5: Isolation-by-distance results for each decade. Comparisons are shown as dots, and a linear regression is shown in black. Mantel test results are given in the respective panel. The samples from Gotland and the Arctic mountains are excluded

#### Figure S6: Genetic sex determination using mlrho. Since bumblebees are haplodiploid, we used the heterozygosity estimates (theta) to determine genetic sex. For each sample, we compare the number of heterozygous sites per kbp with sequencing depth. Historical samples are colored in light gray, modern samples in dark gray. We observe a clear gap between ~0.17 and ~1.38 heterozygous sites per 1000 bp, which we hence describe as the gap between genetic males and females. Specimens morphologically identified as males are shown as squares, females as circles, and those with missing data as rhombuses.

#### Figure S7: AMBER results for all historical samples.

In each plot, the mismatch rate is shown in black dots against the read length. The values are described on the left y-axis. The gray distribution curve indicates the number of reads in thousands, as shown by the right y-axis. The read length cutoff is shown by the dark red vertical line.

**Continuation of Figure S7: AMBER results for all historical samples.**

**Continuation of Figure S7: AMBER results for all historical samples.**

**Continuation of Figure S7: AMBER results for all historical samples.**

**Continuation of Figure S7: AMBER results for all historical samples.**

**Continuation of Figure S7: AMBER results for all historical samples.**

**Continuation of Figure S7: AMBER results for all historical samples.**

**Continuation of Figure S7: AMBER results for all historical samples.**

**Continuation of Figure S7: AMBER results for all historical samples.**
